# Factors Associated with Women's Chronic Disease Management: Associations of Healthcare Frustrations, Physician Support, and Self-Care Needs

**DOI:** 10.1155/2013/982052

**Published:** 2013-10-02

**Authors:** Matthew Lee Smith, Marcia G. Ory, SangNam Ahn, Toni P. Miles

**Affiliations:** ^1^The University of Georgia, College of Public Health, Department of Health Promotion and Behavior, 330 River Road, 315 Ramsey Center Athens, GA 30602, USA; ^2^Texas A&M Health Science Center, School of Rural Public Health, Department of Health Promotion and Community Health Sciences, TAMU 1266, College Station, TX 77843, USA; ^3^The University of Memphis, School of Public Health, Division of Health Systems Management and Policy, Robison Hall 133, Memphis, TN 38152-3530, USA; ^4^The University of Georgia, College of Public Health, Department of Epidemiology and Biostatistics, 255 E. Hancock Avenue, Athens, GA 30602, USA

## Abstract

Previous research emphasizes the importance of reducing healthcare frustrations and enhancing physician supports to help patients engage in recommended healthcare regimens. However, less is known about how these factors are associated with aging women's knowledge about self-care behavior. This study examined the sociodemographics, health indicators, healthcare-related frustrations, and perceptions of physician support associated with middle-aged and older adult females' self-reported need for help to learn how to take better care of their health. Data were analyzed from 287 females with one or more chronic conditions who completed The National Council on Aging (NCOA) Chronic Care Survey. A logistic regression model was developed. Women who were non-White (OR = 2.26, *P* = 0.049) were more likely to need help learning how to better manage their health. Those who had some college education or more (OR = 0.55, *P* = 0.044) and lower healthcare-related frustrations (OR = 0.44, *P* = 0.017) and perceived to have more physician support (OR = 0.49, *P* = 0.033) were less likely to need help learning how to better manage their health. Findings can inform the planning, implementation, assessment, and dissemination of evidence-based self-management programs for middle-aged and older women within and outside of clinical settings.

## 1. Introduction

Over the next several decades, the number of Americans living to advanced ages will increase substantially. Although many individuals will age in relatively good health, a growing number will encounter challenges associated with the burdens of chronic conditions and associated disabilities [1–3]. This is especially so for the large numbers of women who will continue to outlive their male counterparts and likely live those additional years with chronic illnesses requiring day-to-day management [4, 5]. Further, with a dramatic increase of female “baby boomers” with obesity-related chronic conditions, accompanied by reduced fertility rates among this rapidly aging cohort, the additive or multiplicative effects of living with one or more chronic conditions are likely to result in a diminution of (1) individuals' capacity to adequately care for themselves, (2) caregivers to serve as efficient resources, and (3) healthcare providers to give adequate attention and guidance to complex patients with multiple chronic conditions (MCCs) [6].

In line with the millions of older women struggling to manage the symptoms associated with chronic disease, there is growing recognition about the importance of self-care behavior, which is supported by strong epidemiological documentation regarding the positive association of self-care and health outcomes [7–10]. However, this issue transcends women's exposure to and understanding of pertinent information and their development of self-care skills. Older women with chronic conditions also need to assess their surrounding resources to develop the confidence and efficacy necessary to initiate and maintain self-care behavior [11–13]. Self-care behavior are intended to draw upon one's physical, social, and healthcare environments to compensate for, or delay, physical limitations and chronic conditions from progressing into more severe disabilities [14]. Further, self-care skills include (1) identifying strategies that enable older women to adopt and institute appropriate self-care behavior such as getting adequate exercise, eating healthy, or managing medications [15, 16]; (2) interacting with healthcare providers to obtain resources and referrals necessary to manage the progression of chronic conditions [10, 17, 18]; and (3) locating social and community resources to become more educated about their conditions and alleviate the stressors and frustration acting as barriers to self-care behavior [6, 19]. 

While a growing literature has identified general disparities related to self-care among women with chronic conditions based on their race/ethnicity [20–22], socioeconomic status [22–24], and residential rurality [22, 25], the extent to which self-care disparities exist based on these and other sociodemographics requires further investigation. Similarly, the role of education an indicator of socioeconomic status, healthcare access, and health behavior engagement emphasizes its importance when assessing disparities issues [26, 27]. Additional efforts are needed to examine the influence of sociodemographics on self-care skills and behavior among aging women, especially in the presence of perceptions about healthcare-related factors.

Healthcare provider-patient interactions can foster self-care behavior [17] although such interactions can also have a less-recognized negative effect on disease self-management. Women can feel frustrated and helpless when their physicians do not fully explain or clarify the causes of their disease or ways how to best manage their illness [28]. Some patients report not having enough time to address their concerns, or that their physicians simply would not listen to them. Conversely, among patients with diabetes, those who have good communication with their physicians report feeling more involved in decision-making efforts to manage their condition more effectively. Also, patients who report their physicians provided adequate information about their conditions were more likely to better self-manage their illnesses [29]. 

Healthcare professionals often encounter challenges to address their female patients' MCC alongside existing barriers to self-care behavior in their home or community environments. Regardless of the source or cause of these barriers, competing demands on the side of either the patient or provider have potential to create a recursive relationship resulting in disconnect, miscommunication, frustration, and fewer self-care practices [30]. These occurrences may inevitably contribute to decreased health outcomes and rapid chronic disease progression, which highlights the importance of support mechanisms available to the patient.

Women with chronic conditions receive self-care support from various channels. Effective self-care support mechanisms and resources identified to promote self-care behavior include traditional in-person, familial, and community support systems (e.g., support groups and faith-based organizations) [6, 31–35]; however, an emergence in technology-based support mechanisms has been shown to enable individuals with chronic conditions to access and utilize self-care information, despite traditional barriers [18, 36–39]. Further, the active seeking of self-care support and resources has been shown to enhance self-care behavior among individuals with chronic conditions [12, 38, 40]. The preferred and/or utilized support mechanisms differ by population subgroup and type of chronic condition. Evidence shows that racial/ethnic minorities with chronic conditions report increased in-person support mechanisms compared to their non-Hispanic white counterparts [41, 42]. Conversely, those residing in rural areas have shown improvement in their self-care behavior through the use of internet-based support mechanisms [43], which may be critical to overcome traditional challenges associated with geographic isolation, less healthcare resources, and longer travel distances to healthcare services [25, 44].

**Figure 1:**
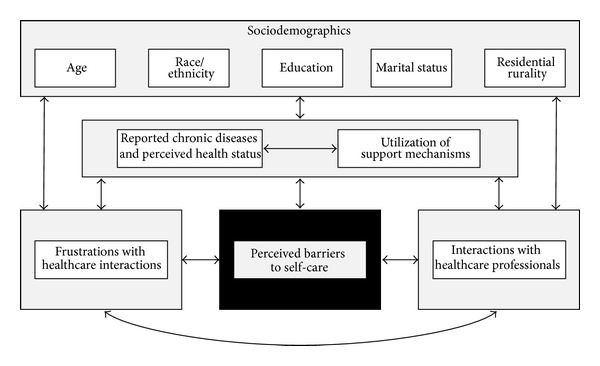
Conceptual framework indicating influences on women's perceived barriers to self-care.

The advancing study of self-care behavior has identified factors influencing the adoption and maintenance of self-care behavior for different populations and people of all ages, with more recent attention paid to lifestyle relative to disease self-care behavior [17, 45, 46]. To advance the translation of research to practice, this secondary data analysis assesses issues surrounding self-care barriers, healthcare-related frustrations, and perceived supports among middle-aged and older adults with one or more chronic conditions. In an effort to further understand the multilevel influences on perceived barriers to self-care, this study will examine the roles of healthcare frustrations and doctor-patient interactions alongside other simultaneously occurring contextual factors (see Figure 1). More specifically, the purposes of this study were to (1) describe sociodemographic variables, health indicators, healthcare-related frustrations, and perceptions of physician support among middle-aged and older adult women with one or more chronic conditions and (2) identify these factors' association with reporting the need to help learning how to take better care of their health among this female population.

## 2. Materials and Methods

### 2.1. Study Participants and Procedures

The National Council on Aging (NCOA), with support from Atlantic Philanthropies and the California HealthCare Foundation (CHCF), commissioned the NCOA Chronic Care Survey, which offers unique insight into the lives of Americans with chronic health conditions [47]. The NCOA Chronic Care Survey is a nationally representative probability survey of 960 community-dwelling men and women aged 44 years and older with at least one chronic condition. Lake Research Partners utilized telephone-based interviewing to collect data using random digit dialing (RDD) sampling techniques, which oversampled those aged 65 and older and Hispanics/Latinos. Telephone interviews were conducted in both English and Spanish. The dataset was weighted by age, race, and region to reflect the overall population of Americans 44+ with chronic condition(s). The margin of sampling error for the total results was ±2.9 percentage points. Margin of error is greater when analyzing smaller subgroups within the sample.

 To be eligible for inclusion in The NCOA Chronic Care Survey, participants had to report having at least one chronic condition at the time of the study. Participants were screened for chronic condition(s) with the following question(s): “Have you ever been told by a doctor, nurse, or other health professional that you have (name of chronic condition)?” Chronic conditions included in the screening process included heart disease, cancer, stroke, diabetes, arthritis, asthma, hypertension or high blood pressure, emphysema, chronic bronchitis, depression, anxiety, and others. Only participants who reported “yes” to at least one of these items were included in the survey.

Of the 960 adults age 44 years and older in this sample, only women were included in study analyses (*n* = 427; 44.5%). Of these women, an additional 140 cases (32.8%) were omitted for incomplete data on variables of interest. More specifically, cases were omitted for incomplete data on rurality (*n* = 48), frustration with the healthcare system (*n* = 25), perceived physician support (*n* = 18), self-reported chronic conditions (*n* = 17), marital status (*n* = 8), using the Internet for general support (*n* = 7), race/ethnicity (*n* = 6), education (*n* = 4), perceived barriers to self-care (*n* = 4), and activity limitations (*n* = 3). The analytic sample for this study contained 287 community-dwelling women, aged 44 years and older who self-reported having at least one chronic condition. When comparing characteristics of women omitted from the study (*n* = 140) with those in the analytic sample (*n* = 287), a significantly larger proportion of the analytic sample was younger (*χ* ^2^ = 4.98, *P* = 0.026), non-Hispanic white (*χ* ^2^ = 4.60, *P* = 0.032), and married (*χ* ^2^ = 11.78, *P* = 0.001). No significant differences were observed based on education or residential rurality.

### 2.2. Data and Measures

#### 2.2.1. Dependent Variable

Participants were asked to self-report their perceived barriers to self-care using an item intended to measure their need for help to *learn how* to take better care of their health. More specifically, participants were asked to rate their level of agreement to the following statement: “I need help learning how to take better care of my health in a way that works for me and my life.” Responses were scored on a 5-point Likert-type scale ranging from “strongly disagree” to “strongly agree.” Based on the frequency distribution, participant responses were then dichotomized into two categories: “disagree” (scored 0) and “agree” (scored 1).

#### 2.2.2. Healthcare Frustrations

Participants were asked to report their frustrations with healthcare interactions using items intended to measure their feelings about repeatedly having to describe their conditions at each doctor visit, the self-care instructions they received from the healthcare provider, the time spent interacting with the healthcare provider, and having a friend or family member attend physician's visits with them. For example, participants were asked to rate their level of agreement to statements like “How often do you feel tired of describing your same conditions and problems every time you go to a hospital or doctor's office?” and “How often do you wish you had a friend or family member who could go to the doctor with you?” Responses were scored using a 3-point Likert-type scale with categories of “never” (scored 0), “occasionally” (scored 1), and “frequently” (scored 2). The Healthcare-Related Frustration Scale (ranging from 0 to 12) was created using these six items. Factor analysis with Varimax rotation was performed to generate this scale. All items loaded on one factor and the items were summed into a single-composite score (*α* = 0.766). Higher scores for the Healthcare-Related Frustration Scale indicate a higher level of frustration with healthcare interactions. Based on the frequency distribution, this scale was converted into tertiles for the analytic purposes. The highest tertile (i.e., representing the highest frustration levels) served as the referent group.

#### 2.2.3. Perceived Physician Support

Participants were asked to self-report the degree to which their physician engages them in treatment-related problem-solving/decision-making, refers them to other healthcare services and professionals, and asks if they understand their medications and associated regimens. For example, participants were asked to rate their level of agreement to statements like “How often does your physician ask for your ideas about how you can take care of your health problems?” and “How often does your physician talk to other doctors and nurses who are taking care of you?” Responses were scored using a 5-point Likert-type scale with categories of “never” (scored 1), “rarely” (scored 2), “occasionally” (scored 3), “frequently” (scored 4), and “always” (scored 5). The Perceived Physician Support Scale (ranging from 6 to 30) was created using these six items. Factor analysis with Varimax rotation was performed to generate this scale. All items loaded on one factor and the items were summed into a single-composite score (*α* = 0.776). Higher scores for the Perceived Physician Support Scale indicate a higher level of perceived physician support. Based on the frequency distribution, this scale was converted into tertiles for the analytic purposes. The lowest tertile (i.e., representing the lowest perceived support levels) served as the referent group.

#### 2.2.4. Health Status Indicators

Participants were asked to self-report aspects of their current health status using items intended to measure activity limitations, the number of prescription medications taken regularly each day, and the number of physician visits in the previous 12 months. Participants were asked “Are you limited in any way in any activities because of physical, mental, or emotional problems?” Responses were scored as “no” (scored 0) or “yes” (scored 1). Participants were asked “How many different prescription medications do you regularly take each day?” Responses ranged from 0 to 26 for this open-ended item. Participants were also asked “In the past 12 months, how many times have you, yourself made a doctor visit?” Responses ranged from 0 to 10 for this open-ended item. 

#### 2.2.5. General Support Perceptions

Participants were asked to self-report their perceptions about their use of the Internet for general support related to managing their health problems. Participants were asked “How much do you rely on the Internet for ongoing help and support with your health problems?” Responses were scored on a 4-point Likert-type scale with categories of “not at all” (scored 0), “a little” (scored 1), “some” (scored 2), and “a lot” (scored 3). Based on the frequency distribution, participant responses were then dichotomized into two categories: “no” (scored 0; indicating that they do not rely on the internet at all) and “yes” (scored 1; indicating that they rely on the internet at least a little). Participants were also asked “How often do you feel you get the help and support you need to improve your health and manage your health problems?” Responses were scored using a 5-point Likert-type scale with categories of “never” (scored 0), “rarely” (scored 1), “occasionally” (scored 2), “frequently” (scored 3), and “always” (scored 4). 

#### 2.2.6. Sociodemographic Characteristics

To identify personal characteristics of these women, sociodemographic variables in this study included age (i.e., 44–64 years, 65+ years); race/ethnicity (i.e., non-Hispanic white, non-White); education level (i.e., high school or less, some college or more); marital status (i.e., unmarried, married); and residential rurality (i.e., urban, suburban, and rural). 

### 2.3. Data Analysis

All statistical analyses were performed using IBM SPSS (version 20). Frequencies were calculated for all major study variables, which were initially examined in relationship to participants' age group (44–64 years, 65+ years) and whether they reported needing help learning how to take better care of their health (yes or no). Pearson's **χ** ^2^ tests were performed to assess the independence between the dependent variable and categorized independent variables. *t*-tests were used to examine mean differences for continuous variables. Logistic regression was performed to examine how sociodemographics, health indicators, perceived support, and frustrations were associated with reporting the need to help learning how to take better care of their health (i.e., not needing help served as the referent group). Odds ratios (OR) with 95% confidence intervals are displayed.

## 3. Results

### 3.1. Sample

Sample characteristics of study participants are presented in Table 1. Of the 287 females participating in this study, 34.5% reported needing help learning how to better care for their health. Over 65% of participants were between the ages of 44 and 64 years and 34.4% were aged 65 years and older. Respondents were disproportionately non-Hispanic white (88.5%), married (69.0%), and had an education level of some college or more (61.7%). Fifty percent of the study population resided in suburban areas, 25.2% resided in urban areas, and 24.8% resided in rural areas. Approximately 33% of participants reported being limited from activities because of physical, mental, or emotional problems. On average, participants reported taking 3.67 (±3.83) medications daily and visiting a physician 3.07 (±1.94) times in the previous 12 months. Over 45% of participants reported relying on the Internet for ongoing help and support to manage their health problems, and 67% of participants reported frequently or always getting the help and support they need to improve their health and manage their health problems.

Compared to women aged 65 years and older, a significantly larger proportion of participants aged 44–64 years had some college education or more (*χ* ^2^ = 7.14, *P* = 0.008) and relied on the Internet for ongoing support to manage their health problems (*χ* ^2^ = 21.15, *P* < 0.001). A significantly larger proportion of participants who reported needing help learning how to better care for their health problems were non-White (*χ* ^2^ = 4.78, *P* = 0.029) and had a high school education or less (*χ* ^2^ = 4.23, *P* = 0.040). 

### 3.2. Healthcare-Related Frustrations

 Healthcare-Related Frustration Scale characteristics are presented in Table 2. Of those who reported healthcare-related frustrations, 16.4% reported frequently wishing their doctor had more time to spend talking to them; 16.0% reported frequently feeling tired of describing their same conditions and problems every time they go to a hospital or doctor's office; 8.7% reported frequently wishing they had a friend or family member who could go to the doctor with them; and 8.3% reported frequently being tired of feeling on their own when it comes to taking care of their health problems. Fewer respondents (5.6%) reported frequently feeling their doctor does not realize what it is really like for them at home trying to take care of their health problems or (5.2%) frequently leaving the hospital or doctor's office feeling confused about what they should do. On average, participants scored 2.56 (±2.69) on the Healthcare-Related Frustration Scale. No significant differences were observed when comparing healthcare-related frustrations by age group. Conversely, when comparing frustrations by whether the participant reported needing help learning how to better care for their health problems, those needing help reported significantly higher scores on the Healthcare-Related Frustration Scale (*t *= −4.79, *P* < 0.001) and higher frustration levels for five of the six individual scale items (*t* = 25.94, *P* < 0.001).

### 3.3. Perceived Physician Support

 Perceived Physician Support Scale characteristics are presented in Table 3. Of those who reported receiving physician support, 50.9% reported their physician always helped them get an appointment they needed; 46.5% reported their physician always asked if they understood their medications when their doctor prescribed them; 15.7% reported their physician always talked to other doctors and nurses who were taking care of them; and another 15.7% reported their physician always made plans to contact them after a visit to see how they were doing. Fewer respondents (13.3%) reported their physician always told them about other people who could help them with their health problems or (13.2%) their physician always asked for their ideas about how they can take care of their health problems. On average, participants scored 18.70 (±5.84) on the Perceived Physician Support Scale. No significant differences were observed when comparing perceived physician support by age group. Conversely, when comparing support by whether the participant reported needing help learning how to better care for their health problems, a significantly smaller proportion of those needing help reported asked for their ideas about how they can take care of their health problems (*χ* ^2^ = 10.48, *P* = 0.033).

### 3.4. Factors Explaining Needing Help Learning How to Take Better Care of Own Health

Table 4 displays the results of the logistic regression analysis explaining factors associated with participants reporting they need help learning how to better care for their health problems (i.e., not needing help served as the referent group). Participants who were non-White were significantly more likely to report needing help learning how to better care for their health problems (compared to non-Hispanic whites, OR = 2.26, *P* = 0.049), whereas those with some college or more were significantly less likely to report needing help learning how to better care for their health problems (compared to those with high school or less education, OR = 0.55, *P* = 0.044). Compared to participants with the highest level (tertile) of healthcare-related frustrations, those with middle (OR = 0.17, *P* < 0.001) and lowest (OR = 0.44, *P* = 0.017) frustration levels were significantly less likely to report needing help learning how to better care for their health problems, respectively. Compared to participants with the lowest level (tertile) of perceived physician support, those with the highest level of perceived support were significantly less likely to report needing help learning how to better care for their health problems (OR = 0.49, *P* = 0.033).

## 4. Discussion

Despite our concerns that the majority of older women would likely experience self-care problems [48–50], our analyses revealed that only about one-third of the women in our sample reported needing help learning how to take better care of their chronic conditions. But consistent with health disparities in the epidemiology of chronic illnesses [22, 24], persistent health disparities related to self-care behavior were noted among minorities and those with less education. The rural healthcare disparity often reported in other studies [25, 51–53] was not observed in this study, nor were unmarried women disadvantaged relative to their married counterparts. These findings suggest middle-aged and older women in this sample were comparable in terms of having a variety of social supports for learning how to take care of their health. 

Additionally, despite the previous assumption that older women might be disadvantaged relative to younger baby-boomers, no significant differences in help needed based on age group were reported. Similarly, our proxy measures for disease magnitude and severity (i.e., number of medications, physician visits, and limitations in activities) did not differentiate those needing help. However, the existence of an age-based digital divide was observed [54–56], with older women less likely to use online/technology as a resource for chronic condition self-management. Further investigation is needed to examine differences in the types of methods/strategies in which these older women engage when caring for their health conditions outside of the healthcare setting. 

This study helps elucidate the complex relationships among contextual factors, healthcare frustrations, and patient-provider interactions and support and points to potential opportunities for intervention. The lack of a significant relationship between age and healthcare-related frustrations or perceptions about physician support may be attributed to the fact that the sample was selected based on the presence of chronic conditions. However, the lack of a significant relationship is consistent with the fact that participation in community-based disease self-management programs is not limited to those of only older ages; rather, program enrollment is based on the participant's chronic disease status [57]. 

This study reveals two strong modifiable correlates of women needing help learning how to care for their chronic conditions, even after controlling for sociodemographic and health status indicators in multivariate analyses: healthcare-related frustrations and perceived physician support. The majority of identified frustrations were significantly related to middle-aged and older women's perceptions of needing help learning how to care for their health, which is supported in other studies [8, 16, 58, 59]. The recent emphasis on patient-centered care and medical homes [60] is designed to help reduce such frustrations and hence can be expected to help boost women's self-efficacy to care for their own health conditions. Additionally, perceived physician support was another significant factor for knowing how to self-manage chronic conditions, especially in terms of patient activation as exemplified by wanting physicians to “ask for your ideas about how you can take care of your health problems.” This reinforces previous research about the importance of perceived physician support for motivating patients to engage in healthier lifestyles and recommended medical regimens [9, 31, 61, 62]. From the health professional point of view, fostering beliefs of patient support can be accomplished through continuing education units (CEUs) or expanded emphasis during medical school training to develop strategies in which clinicians can engage and implement to be supportive, listen to their patients, and solicit their patients' thoughts so they have an active role in their healthcare team [29, 63]. From the patient point of view, widely available evidence-based self-management programs include elements within their curricula to teach older women how they can more effectively communicate with their healthcare providers [64]. Considering perspectives from each side of the healthcare interaction is essential to improve self-management both within and outside of the healthcare setting, which has implications for reducing disease mismanagement, unnecessary healthcare utilization (e.g., emergency room use), and medical costs. 

## 5. Limitations

This secondary dataset did not contain all variables necessary to fully contextualize barriers and challenges associated with chronic disease self-care behavior among this aging population. However, this national study contained many variables of interest to address the current research gaps in knowledge about associations of healthcare frustrations, physician support, and self-care needs to chronic disease management. 

Substantial numbers of participants were lost due to incomplete data on particular scale items, resulting in an analytical sample that was systematically different from the full sample (e.g., younger, married, and more white) and potentially limited the generalizability of study findings. This is especially true in that the reduction of cases in the analytic sample reduced the potential proportion of older adults in the study from 42.1% to 33.4%, which may especially influence generalizability of findings to populations aged 65 years and older. Study participants reported a variety of chronic conditions, but subanalyses based on disease type were not performed because the sample size was inadequate to make such comparisons. Recognizing that needed self-care behavior may differ based on women's particular condition diagnoses, disease stage, and the number of comorbidities in which they are diagnosed, future studies should strive to compare about barriers to self-care, perceived physician support, and frustrations with the healthcare system by their chronic condition profile. Further, because women's ability to cope with and adjust to their disease self-care may differ based on their available resources and socioeconomic status, future studies should examine such variables to determine their relationship with self-care disparities.

Another study limitation reducing the ability to widely generalize findings to the greater female community was the self-report and cross-sectional nature of these data. However, the sample was derived from random digit dialing and included items that deeply explore the challenges and frustrations with chronic condition self-management, which are not typically seen in other studies that investigate correlations between self-care behavior and other healthcare or physical health indicators. Thus, this study contributes to a fuller understanding of the complex interrelationships that exist between self-care strategies, provider-patient interactions, and policies/programs in community contexts.

## 6. Conclusion

The current study adds to the existing science base by examining barriers to self-care with a new lens, exploring healthcare-related frustrations and perceptions of physician support, and how these perceptions relate to various life domains, including diverse health status and sociodemographic contexts. Identifying these common and unique challenges and correlates of these challenges, with a representative national population, advance our current knowledge about self-care issues among middle-aged and older women. Learning more about healthcare-related frustrations of and self-management supports utilized by middle-aged and older women has the potential to help program deliverers, healthcare providers, and health agencies provide the services and resources that women with chronic conditions want and think are helpful. Findings from this investigation has potential to inform and guide modifications in the implementation and dissemination of evidence-based programs for older women to better match individuals with programs that meet their needs.

## Figures and Tables

**Table 1:** Sample characteristics by age and needing help learning HOW to take better care of own health.

	Total (*n* = 287)	Age group				Need help learning How to care for health			
		44 to 64 (*n* = 189)	65+ (*n* = 98)	*χ* ^2^ or *t*	*P*	No (*n* = 188)	Yes (*n* = 99)	*χ* ^2^ or *t*	*P*
Age									
44 to 64 years	65.6%	—	—	—	—	64.4%	68.0%	0.38	0.536
65+	34.4%	—	—			35.6%	32.0%		
Race/ethnicity									
Non-Hispanic white	88.5%	86.8%	91.8%	1.63	0.202	91.5%	82.8%	4.78	0.029
Non-White	11.5%	13.2%	8.2%			8.5%	17.2%		
Education									
High school or less	38.3%	32.8%	49.0%	7.14	0.008	34.0%	46.5%	4.23	0.040
Some college or more	61.7%	67.2%	51.0%			66.0%	53.5%		
Marital status									
Unmarried	31.0%	28.0%	36.7%	2.28	0.131	27.8%	37.0%	2.57	0.109
Married	69.0%	72.0%	63.3%			72.2%	63.0%		
Residential rurality									
Urban	25.2%	17.4%	8.0%	0.33	0.847	23.5%	28.3%	1.95	0.376
Suburban	50.0%	48.9%	52.0%			49.2%	51.5%		
Rural	24.8%	24.7%	24.5%			27.3%	20.2%		
Limited activities for any reason (physically/mentally/emotionally)									
No	67.4%	64.7%	72.4%	1.75	0.186	71.3%	60.0%	3.78	0.052
Yes	32.6%	35.3%	27.6%			28.7%	40.0%		
Get help and support to improve health and manage health problems									
Never	5.6%	6.3%	4.0%	1.27	0.866	5.9%	5.0%	7.98	0.092
Rarely	9.7%	9.5%	10.1%			9.0%	11.0%		
Occasionally	17.7%	11.8%	5.9%			14.9%	23.0%		
Frequently	31.9%	21.5%	10.4%			29.8%	36.0%		
Always	35.1%	33.3%	38.4%			40.4%	25.0%		
Rely on internet for ongoing support to manage health problems									
No	54.7%	45.0%	73.5%	21.15	<0.001	53.7%	56.6%	0.21	0.646
Yes	45.3%	55.0%	26.5%			46.3%	43.4%		
Number of medications taken daily	3.67 (±3.83)	3.64 (±4.18)	3.73 (±3.08)	−0.20	0.843	3.58 (±3.77)	3.84 (±3.96)	−0.56	0.574
Number of physician visits in the previous 12 months	3.07 (±1.94)	3.10 (±2.00)	3.00 (±1.83)	0.41	0.684	3.00 (±1.97)	3.18 (±1.89)	−0.75	0.452

**Table 2:** Healthcare frustrations by age and needing help learning HOW to take better care of own health.

Healthcare-related frustration scale items (*α* = 0.766)	Total (*n* = 287)	Age group				Need help learning how to care for health			
		44 to 64 (*n* = 189)	65+ (*n* = 98)	*χ* ^2^ or *t*	*P*	No (*n* = 188)	Yes (*n* = 99)	*χ* ^2^ or *t*	*P*
Wish your doctor had more time to spend talking with you									
Never	59.1%	56.8%	62.6%	1.47	0.479	67.4%	43.4%	18.54	<0.001
Occasionally	24.5%	24.7%	24.2%			21.9%	29.3%		
Frequently	16.4%	18.4%	13.1%			10.7%	27.3%		
Feel tired of describing your same conditions and problems every time you go to a hospital or doctor's office									
Never	59.4%	59.8%	59.2%	2.01	0.367	67.6%	44.0%	16.42	<0.001
Occasionally	24.7%	22.8%	28.6%			21.3%	31.0%		
Frequently	16.0%	17.5%	12.2%			11.2%	25.0%		
Wish you had a friend or family member who could go to the doctor with you									
Never	76.0%	76.2%	76.5%	0.06	0.969	80.9%	66.7%	7.40	0.025
Occasionally	15.3%	14.8%	15.3%			11.7%	22.2%		
Frequently	8.7%	9.0%	8.2%			7.4%	11.1%		
Tired of feeling on your own when it comes to taking care of your health problems									
Never	68.1%	66.8%	70.7%	1.93	0.381	72.9%	59.0%	5.80	0.055
Occasionally	23.6%	25.8%	19.2%			20.2%	30.0%		
Frequently	8.3%	7.4%	10.1%			6.9%	11.0%		
Feel that your doctor does not realize what it is really like for you at home trying to take care of your health problems									
Never	71.4%	70.4%	72.7%	1.84	0.398	78.2%	58.6%	20.81	<0.001
Occasionally	23.0%	22.8%	24.2%			14.9%	38.4%		
Frequently	5.6%	4.5%	1.0%			6.9%	3.0%		
Leave the hospital or a doctor's office and feel confused about what you should do									
Never	71.0%	70.9%	70.7%	0.01	0.994	79.7%	54.5%	19.86	<0.001
Occasionally	23.8%	23.8%	24.2%			16.6%	37.4%		
Frequently	5.2%	5.3%	5.1%			3.7%	8.1%		
Healthcare-related frustration tertiles (higher scores indicate more frustration)									
Lowest tertile	30.30%	30.00%	30.60%	1.89	0.388	38.30%	15.20%	25.94	<0.001
Middle tertile	47.00%	44.70%	51.00%			30.70%	16.40%		
Highest tertile (referent group)	22.60%	25.30%	18.40%			14.90%	37.40%		
Healthcare-related frustration scale (ranging from 0 to 12)	2.55 (±2.69)	2.64 (±2.79)	2.39 (±2.50)	0.75	0.452	2.00 (±2.47)	3.60 (±2.80)	−4.79	<0.001

**Table 3:** Perceived physician support by age and needing help learning HOW to take better care of own health.

Perceived physician support scale items (*α* = 0.776)	Total (*n* = 287)	Age group				Need help learning how to care for health			
		44 to 64 (*n* = 189)	65+ (*n* = 98)	*χ* ^2^ or *t*	*P*	No (*n* = 188)	Yes (*n* = 99)	*χ* ^2^ or *t*	*P*
Help you get appointments that you need									
Never	12.9%	14.3%	10.2%	4.66	0.324	16.0%	7.1%	8.50	0.075
Rarely	5.9%	3.8%	2.1%			4.8%	8.1%		
Occasionally	12.9%	9.1%	3.8%			10.1%	18.2%		
Frequently	17.4%	12.9%	4.5%			17.6%	17.2%		
Always	50.9%	30.7%	20.2%			51.6%	49.5%		
Ask if you understand your medications when your doctor prescribes them									
Never	15.3%	15.8%	14.1%	1.85	0.763	17.6%	11.0%	7.30	0.121
Rarely	6.6%	7.4%	5.1%			6.4%	7.0%		
Occasionally	14.2%	9.7%	4.5%			10.6%	21.0%		
Frequently	17.4%	10.4%	7.3%			18.6%	15.0%		
Always	46.5%	46.3%	46.5%			46.8%	46.0%		
Talk to other doctors and nurses who are taking care of you									
Never	25.4%	25.4%	25.5%	4.08	0.395	28.3%	20.0%	4.79	0.309
Rarely	15.0%	11.5%	3.5%			12.3%	20.0%		
Occasionally	26.8%	24.3%	31.6%			26.2%	28.0%		
Frequently	17.1%	11.8%	5.2%			16.6%	18.0%		
Always	15.7%	9.8%	5.9%			16.6%	14.0%		
Make plans to contact you after a visit to see how you are doing									
Never	26.8%	28.6%	24.5%	2.80	0.591	28.3%	24.0%	4.25	0.373
Rarely	15.3%	10.8%	4.5%			13.4%	19.0%		
Occasionally	25.1%	16.4%	8.7%			27.8%	20.0%		
Frequently	17.1%	9.4%	7.3%			16.0%	19.0%		
Always	15.7%	10.5%	5.2%			14.4%	18.0%		
Tell you about other people who can help you with your health problems									
Never	30.1%	19.4%	10.4%	5.06	0.282	30.5%	29.3%	3.56	0.469
Rarely	11.5%	6.6%	5.2%			12.3%	10.1%		
Occasionally	30.1%	18.7%	11.1%			30.5%	29.3%		
Frequently	15.0%	11.8%	3.5%			12.3%	20.2%		
Always	13.3%	9.7%	3.8%			14.4%	11.1%		
Ask for your ideas about how you can take care of your health problems									
Never	29.2%	19.2%	9.8%	2.66	0.616	33.0%	22.0%	10.48	0.033
Rarely	13.2%	9.8%	3.5%			11.7%	16.0%		
Occasionally	23.6%	14.3%	9.4%			18.6%	33.0%		
Frequently	20.8%	14.7%	6.3%			22.3%	18.0%		
Always	13.2%	12.2%	14.4%			14.4%	11.0%		
Perceived physician support tertiles (higher scores indicate more support)									
Highest tertile	25.40%	27.50%	21.40%	1.93	0.381	27.10%	22.20%	1.10	0.577
Middle tertile	41.50%	41.80%	40.80%			41.50%	41.40%		
Lowest tertile (referent group)	33.10%	30.70%	37.80%			31.40%	36.40%		
Perceived physician support scale (ranging from 6 to 30)	18.70 (±5.84)	18.52 (±5.83)	19.03 (±5.87)	−0.70	0.482	18.53 (±6.06)	19.01 (±5.43)	−0.67	0.505

**Table 4:** Factors associated with needing help learning HOW to take better care of own health.

	OR	*P*	95% CI	
			Lower	Upper
Age 44 to 64 years	1.00	—	—	—
Age 65+	0.79	0.437	0.43	1.43
Non-Hispanic white	1.00	—	—	—
Non-White	2.26	0.049	1.00	5.09
High school or less	1.00	—	—	—
Some college or more	0.55	0.044	0.31	0.98
Married: no	1.00	—	—	—
Married: yes	0.96	0.889	0.53	1.74
Urban	1.00	—	—	—
Suburban	1.72	0.178	0.78	3.79
Rural	1.87	0.074	0.94	3.73
Limited activities for any reason: no	1.00	—	—	—
Limited activities for any reason: yes	1.07	0.847	0.56	2.02
Get help and support to manage health problems: never	1.00	—	—	—
Get help and support to manage health problems: rarely	1.23	0.765	0.32	4.64
Get help and support to manage health problems: occasionally	2.11	0.156	0.75	5.90
Get help and support to manage health problems: frequently	1.61	0.260	0.70	3.66
Get help and support to manage health problems: always	1.62	0.171	0.81	3.23
Rely on internet for ongoing support to manage health problems: no	1.00	—	—	—
Rely on internet for ongoing support to manage health problems: yes	0.94	0.848	0.52	1.70
Number of medications taken daily	0.98	0.695	0.91	1.07
Number of physician visits in the previous 12 months	0.93	0.428	0.79	1.11
Healthcare-related frustration scale: highest tertile	1.00	—	—	—
Healthcare-related frustration scale: middle tertile	0.17	<0.001	0.07	0.41
Healthcare-related frustration scale: lowest tertile	0.44	0.017	0.22	0.86
Perceived physician support scale: lowest tertile	1.00	—	—	—
Perceived physician support scale: middle tertile	0.50	0.081	0.23	1.09
Perceived physician support scale: highest tertile	0.49	0.033	0.25	0.94
